# Beyond SINS: A Critical Review of Biomechanical, Microstructural, and Radiomic Biomarkers for Predicting Fracture Risk in Spinal Metastases

**DOI:** 10.3390/diagnostics16121835

**Published:** 2026-06-13

**Authors:** An Sen Tan, Calvin Kai En Tjio, Jonathan Jiong Hao Tan, Naresh Kumar, Wilson Ong, Shuliang Ge, Yi Liang Tan, Eric Fang, Balamurugan A. Vellayappan, James Thomas Patrick Decourcy Hallinan

**Affiliations:** 1 Department of Diagnostic Imaging, National University Hospital, 5 Lower Kent Ridge Rd, Singapore 119074, Singapore; calvin.tjio@mohh.com.sg (C.K.E.T.); wilson.ong@mohh.com.sg (W.O.); shuliang_ge@nuhs.edu.sg (S.G.); yi_liang_tan@nuhs.edu.sg (Y.L.T.); eric_km_fang@nuhs.edu.sg (E.F.); james_hallinan@nuhs.edu.sg (J.T.P.D.H.); 2 National University Spine Institute, Department of Orthopaedic Surgery, National University Hospital, 1E Lower Kent Ridge Road, Singapore 119228, Singapore; jonathan_jh_tan@nuhs.edu.sg (J.J.H.T.); dosksn@nus.edu.sg (N.K.); 3 Department of Radiation Oncology, National University Hospital, 5 Lower Kent Ridge Rd, Singapore 119074, Singapore; bala_vellayappan@nuhs.edu.sg; 4 Department of Diagnostic Radiology, Yong Loo Lin School of Medicine, National University of Singapore, 10 Medical Drive, Singapore 117597, Singapore

**Keywords:** spinal metastases, spinal instability neoplastic score, pathologic fracture, spinal stability, radiation therapy, radiomics, biomechanical modelling, machine learning

## Abstract

**Background/Objectives:** Although the Spinal Instability Neoplastic Score (SINS) is widely used to estimate spinal metastases fracture risk and guide decisions on stabilisation procedures, prior studies have demonstrated mixed results. Patients with the same score exhibit clinically heterogeneous outcomes, with some SINS criteria correlating less well with the estimated fracture risk than others. There are also barriers to implementation such as the time burden required for manual calculation and interobserver variability associated with qualitative morphological criteria. SINS also lacks sensitivity for detecting latent structural compromise in treatment-naive patients and those susceptible to the iatrogenic effects of stereotactic body radiation therapy. This review aims to evaluate emerging imaging, biomechanical, and microstructural markers with the potential to improve fracture risk stratification and prognostication for spinal oncology patients. **Methods:** We synthesise evidence across three innovative frontiers: (1) biomechanical modelling, including CT-derived finite element analysis and failure-load pattern models; (2) radiomics, utilizing radiomics features from radiological imaging to develop a predictive model; and (3) microstructural MRI biomarkers, exploring the translatability of the Vertebral Bone Quality score, fat fraction, and paraspinal muscle atrophy from osteoporosis to the metastatic spine. **Results:** Emerging biomechanical, radiomic and microstructural imaging markers show potential in addressing some limitations of traditional SINS criteria for fracture risk stratification across the spinal oncology treatment continuum, from initial diagnosis to post-radiation surveillance, thereby facilitating more precise risk assessment. However, current evidence remains largely retrospective and heterogeneous, and further validation is required before clinical adoption. **Conclusions:** We propose a framework that shifts the paradigm from conventional morphological scoring toward a multiparametric assessment of spinal stability.

## 1. Introduction

The spine is the most frequent site of skeletal metastasis, affecting approximately 30% to 70% of patients with stage 4 cancer [1]. A devastating complication of this metastatic involvement is the development of pathological vertebral compression fractures (VCF), which occur in 10% to 30% of these patients [2,3]. These fractures frequently cause intractable pain, spinal deformity, and neurological compromise, significantly degrading each patient’s quality of life and autonomy. As systemic therapies and stereotactic body radiation therapy (SBRT) extend patient survival, the preservation of locomotive function and the prevention of skeletal-related events have become critical components of oncological care [3].

To standardise the assessment of tumour-related instability, the Spine Oncology Study Group introduced the Spinal Instability Neoplastic Score (SINS) in 2010 [4]. SINS aggregates six clinical and radiographic components: location, pain, bone lesion type, spinal alignment, vertebral body collapse, and posterolateral involvement, to classify lesions as stable (score 0–6), potentially unstable (7–12), or unstable (13–18). While SINS has successfully established a common language among specialists and demonstrates substantial interobserver reliability for binary stable/unstable categorizations [5], its clinical precision remains limited. A significant proportion of patients fall into the “indeterminate” category (scores 7–12), where treatment guidelines are ambiguous and rely heavily on subjective specialist experience [1]. Furthermore, SINS relies on qualitative morphological criteria that do not fully account for key mechanical determinants governing structural failure. These include the three-dimensional distribution of vertebral material properties or the complex interactions between tumours and the trabecular microarchitecture [2,6]. Consequently, SINS has demonstrated only moderate accuracy in predicting VCF, particularly in the setting of post-radiation fractures where iatrogenic structural compromise is a concern [5].

To bridge the gap between qualitative scoring and the physical reality of spinal failure, research has pivoted toward quantitative biomarkers. High-fidelity biomechanical modelling, specifically subject-specific Finite Element Analysis (FEA) derived from quantitative computed tomography (QCT), has emerged as a method validated in multiple studies for estimating vertebral strength. Unlike clinical scoring systems, FEA can simulate physiological loading conditions to predict failure loads with high correlation to experimental data. These models allow for the granular assessment of how lesion size, type (lytic vs. blastic), and precise location within the individual vertebra—factors often simplified in SINS—specifically degrade structural integrity. Recent computational studies indicate that baseline bone strength and lesion size are critical predictors of fracture risk, often outperforming density-based measures alone [7].

Parallel to biomechanical advancements, the field of radiomics offers a non-invasive method to extract high-dimensional quantitative features from standard medical imaging that may not be apparent to the human eye. Machine learning models incorporating radiomic features from pretreatment CT and magnetic resonance imaging (MRI) have demonstrated the ability to predict VCF risk with greater sensitivity and specificity than clinical features or SINS alone, particularly in patients undergoing SBRT [8,9]. These radiomic signatures capture intrinsic tissue heterogeneity and microstructural irregularities that precede macroscopic structural failure.

This narrative review critically evaluates the evidence “Beyond SINS,” synthesizing emerging data in the three promising frontiers of biomechanical modelling, microstructural assessment, and machine learning methods along with radiomics. We propose a framework that shifts the paradigm from conventional morphological scoring toward a multiparametric assessment of spinal stability, aiming to assist precise, personalised interventions for patients with metastatic spinal disease.

## 2. Methods

### Literature Search Strategy and Selection Criteria

**Figure 1:**
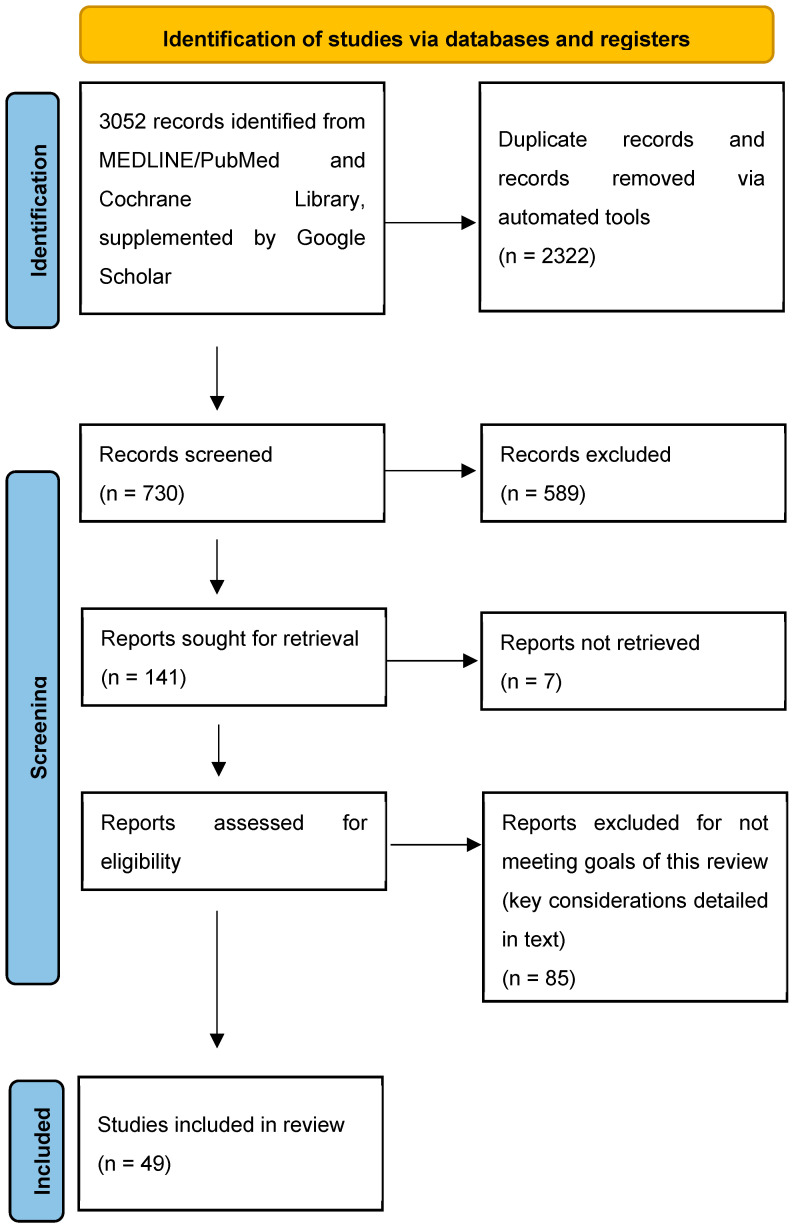
Flowchart detailing literature selection process.

A literature search (Figure 1) was conducted to identify relevant studies evaluating quantitative, computational, and microstructural methods for assessing spinal stability and fracture risk in the context of spinal metastases. We queried electronic databases including PubMed/MEDLINE and the Cochrane Library, supplemented by Google Scholar for articles published between January 2010 (the year SINS was introduced) and April 2026. The core search strategy utilised Boolean combinations of the following keywords and Medical Subject Headings (MeSH): (“spinal metastases” OR “spinal neoplasm”) AND (“fracture risk” OR “spinal instability”) AND (“Spinal Instability Neoplastic Score” OR “SINS” OR “finite element analysis” OR “biomechanical modelling” OR “radiomics” OR “machine learning” OR “vertebral bone quality” OR “MRI biomarkers”). Inclusion criteria were restricted to original research, computational modelling studies, review articles and meta-analyses published in the English language. Case reports, editorials, and studies lacking quantitative fracture prediction metrics were excluded.

The database search yielded a total of 3052 records. Automated tools removed 2322 duplicate records and/or those not meeting the inclusion criteria. Two independent investigators screened titles and abstracts of the remaining 730 records for general relevance, resulting in the exclusion of 589 articles. The remaining 141 articles were sought for full-text assessment. To serve the goals of this narrative review, we prioritised recent investigations (predominantly from the last five years) that either directly compared emerging multiparametric models to traditional SINS criteria or demonstrated novel predictive capabilities in high-risk cohorts. Particular emphasis was placed on investigations evaluating iatrogenic fractures following SBRT. Following assessment, 49 publications were ultimately selected to provide a targeted conceptual synthesis of the current literature.

## 3. The Current Standard and Its Limitations: The Spinal Instability Neoplastic Score (SINS)

### 3.1. Overview of SINS

SINS incorporates five radiographic variables and one clinical factor: global spinal location, mechanical pain, bone lesion quality, spinal alignment, degree of vertebral body collapse, and posterolateral spinal element involvement [4]. Each component receives a weighted numerical value, yielding a cumulative score ranging from 0 to 18. Based on this total, lesions are stratified into three distinct biomechanical categories: stable (0 to 6), potentially unstable or indeterminate (7 to 12), and unstable (13 to 18). Current multidisciplinary consensus guidelines generally recommend surgical consultation for any patient presenting with a score of 7 or greater to evaluate the need for stabilisation [10]. This classification was designed to provide a common lexicon among oncologists, radiologists, and spine surgeons, thereby facilitating more uniform reporting and clinical triage.

### 3.2. Interobserver Variability and Subjectivity

While the morphological scoring system provides a structured communication tool, empirical studies evaluating its interobserver reliability suggest inconsistent precision across its individual components. A comprehensive meta-analysis evaluating the accuracy and precision of the criteria demonstrated that the overall reliability for assigning patients into binary stable and unstable categories achieved substantial agreement. However, the individuals variables showed significant variability [5]. Anatomical location has consistently demonstrated near-perfect reliability, whereas radiographic spinal alignment, vertebral body collapse, and posterolateral involvement generally achieve only moderate agreement [5]. The qualitative assessment of bone lesion matrix quality remains a persistent clinical challenge. Multiple independent investigations have reported only fair interobserver reliability for characterizing lesions as lytic, blastic, or mixed, with kappa values falling at or below 0.30 [5,11,12]. Evaluator clinical background also appears to influence scoring consistency. An evaluation across medical specialties demonstrated that agreement on the final score was fair among non-spine surgeons and less experienced spine surgeons, compared to substantial agreement among highly experienced spinal oncology surgeons [12]. This discrepancy likely reflects the subjective nature of several morphological criteria. The lack of quantitative definitions for parameters such as alignment and collapse necessitates a degree of clinical judgment, which can limit reproducibility when the tool is broadly applied by clinicians in routine clinical settings.

### 3.3. Disproportionate Predictive Value of Individual SINS Criteria

Although SINS weights its six components similarly, quantitative evaluations suggest that these criteria have unequal predictive power for forecasting VCF. Pre-existing vertebral body collapse and the presence of lytic bone lesions consistently emerge as among the most significant independent predictors of structural failure [5,13]. A retrospective analysis by Shi et al. demonstrated that lytic tumour composition was highly predictive of new or worsening fractures, yielding a hazard ratio of 3.70 (95% CI of 1.91 to 7.17; *p* = 0.0001) [13]. Similarly, Gui et al. reported an odds ratio of 2.48 (95% CI of 1.19 to 5.17; *p* = 0.02) for lytic lesions predicting fractures following SBRT. A 2021 meta-analysis by Kim et al. corroborated these structural parameters, reporting a moderate relationship between the degree of initial vertebral body collapse and subsequent fracture, with a correlation coefficient of 0.333 (*p* < 0.001) [5]. The authors noted a nuanced relationship where vertebrae with less than 50% collapse experienced higher subsequent fracture rates (39%) than those with greater than 50% collapse (27%). Extensively compressed vertebrae likely have limited remaining cancellous volume to undergo further height loss, accounting for this paradoxical stabilisation effect.

Other factors display highly variable correlations with fracture outcomes depending on the population analysed. Two articles examined large single institution datasets and reported conflicting findings regarding the presence of mechanical pain and spinal malalignment. Kowalchuk et al. found that both pain and spinal alignment were significantly associated with an increased risk of fracture for each point added in their respective grading categories [14]. Shi et al. similarly identified alignment other than normal as a significant predictor, reporting a hazard ratio of 2.35 (95% CI of 1.23 to 4.50; *p* = 0.01) [13]. Conversely, the aforementioned meta-analysis found negligible relationships between alignment and fracture incidence, likely because extreme subluxation presents too rarely in radiation cohorts to achieve statistical significance with Kim et al. identifying only two such cases (of score 4) in their pooled data of over 1200 lesions. The predictive utility of mechanical pain also remains debated. While a retrospective analysis from the Dana-Farber Cancer Institute found mechanically painful lesions highly predictive of progression to a new or worsening fracture [13], the comprehensive meta-analysis by Kim et al. reported negligible relationships between alignment and fracture incidence. The authors found that patients with occasional pain showed a similar fracture incidence to those who were entirely pain free [5]. This is potentially explained by the observation that scoring this variable relies heavily on accurately distinguishing mechanical symptoms from generalised oncological pain, frequently leading to inconsistent documentation across institutions.

Selection biases inherent in retrospective studies also warrant caution when interpreting the lack of predictive value for certain severe morphological criteria. Patients presenting with extreme instability, such as complete vertebral collapse or high-grade malalignment, frequently undergo surgical stabilisation prior to receiving radiotherapy. This routine clinical triage removes the most severe cases from radiotherapy databases, potentially reducing the statistical power of alignment and collapse in analyses of irradiated cohorts. Consequently, while cumulative scores remain helpful for general triage, individual factors directly governing load bearing capacity may correlate more closely with the true risk of fracture.

### 3.4. The “Indeterminate” Grey Zone

The management of patients scoring between 7 and 12 remains one of the most significant clinical challenges in spinal oncology. Retrospective analyses indicate that approximately 60% of cases fall into this indeterminate range [15,16]. While the morphological criteria reliably predict extreme cases of stability or instability, they provide uncertain surgical indications for intermediate scores.

A systematic review examining clinical outcomes in this specific cohort highlighted wide variations in failure rates of non-operative management ranging from 4.0–25.5% and occasionally necessitating salvage spinal surgery [10]. This underscores the clinical heterogeneity of patients grouped within this indeterminate category.

Prior retrospective analyses evaluating this subset have utilised the incidence of surgical instrumentation as a measure of instability. These suggest the presence of distinct subgroups; patients with scores of 10 to 12 are significantly more likely to receive spinal instrumentation compared to patients scoring 7 to 9. Lenschow and colleagues found that 85.9% of patients with scores of 10 to 12 underwent instrumentation, compared to 62.9% of those scoring 7 to 9 [17]. Similarly, an analysis by Pennington et al. reported that within the potentially unstable grey zone, 79% of patients undergoing instrumentation scored 10 to 12, whereas only 11% scored between 7 and 9 [18]. A cutoff score of 9 or 10 frequently serves as a practical threshold for deciding on spinal instrumentation [10]. Mahakul et al. utilised a score of 9 as the cutoff for instrumentation, achieving a predictive area under the curve (AUC) receiver operator characteristic value of 0.79 (95% CI of 0.67 to 0.91, *p*-value not reported) with a sensitivity of 68% (95% CI of 50.2 to 82.0%, *p*-value not reported) and a specificity of 74% (95% CI of 51.6 to 89.8%, *p*-value not reported) [19]. Meanwhile, Pennington et al. identified a score of 10 as the threshold above which patients had greater than 50% odds of undergoing stabilisation [18].

Relying on instrumentation rates as a surrogate endpoint of instability introduces a degree of bias, as broader systemic factors and multidisciplinary treatment selections often influence operative outcomes rather than isolated vertebral mechanics. For instance, structurally stable vertebrae with low morphological scores occasionally require instrumented fusion following iatrogenic destabilisation from decompressive laminectomies for epidural disease. Conversely, highly unstable vertebrae in moribund patients with poor overall survival prognoses are often managed conservatively.

Overall, the heterogeneity in outcomes within this grey zone demonstrates the inadequacy of relying exclusively on qualitative morphological criteria for clinical decision making.

### 3.5. SINS in the SBRT Era

SBRT achieves excellent local tumour control but increases the risk of subsequent VCF [20]. Conventional morphological criteria have shown inconsistent performance in predicting these iatrogenic fractures across different patient populations.

A meta-analysis in 2021 including 7 retrospective studies (totalling 1269 spinal segments) provides a reasonable estimate of SINS’s performance in this respect. The authors evaluated cutoff score of 7 for predicting post-radiotherapy fractures, reporting a pooled sensitivity of 79.0% (95% CI of 72.3% to 84.3%) alongside a relatively low pooled specificity of 54.6% (95% CI of 46.2% to 62.7%) [5]. In contrast, a more recent investigation by Gui et al. evaluated a machine learning based classification model that combined clinical and radiomic features. This model achieved an AUC value of 0.878 (95% CI of 0.832 to 0.924), substantially outperforming a standalone model including SINS features alone which yielded an AUC of only 0.579 (95% CI 0.496 to 0.663) [21].

A significant methodological limitation accounting for this variable performance is the widespread use of composite endpoints. When evaluating the predictive utility of baseline morphological scoring for post-radiation structural failure, investigators frequently combine the occurrence of incident fractures in previously intact vertebrae with the progressive collapse of vertebrae containing pre-existing fractures [5,8,21]. The mechanical failure of an intact cortex represents a clinically different scenario than the continued trabecular degradation of an already compromised vertebra.

The SINS criteria remain a useful descriptive tool for facilitating multidisciplinary communication of baseline morphology. However, these findings highlight a critical need for additional fracture risk assessment tools within the modern SBRT era.

### 3.6. Overcoming Implementation Barriers

Manual calculation of the morphological criteria is time-intensive and difficult to scale within high-volume clinical workflows. To address these constraints, recent efforts have focused on integrating artificial intelligence, including large language models (LLMs), to automate the scoring process [22,23]. Early automation attempts used artificial intelligence systems that improved reproducibility but still relied on manual image segmentation, highlighting a persistent need for fully automated clinical tools [20]. Recent investigations have demonstrated the utility of natural language processing to efficiently extract necessary data from unstructured electronic medical records and imaging reports. An evaluation of a privacy-preserving LLM showed substantial improvements in triage efficiency, where unassisted manual calculation by clinicians required a median time of 83 s, whereas LLM-assisted scoring reduced this time to 60 s [23]. When generating scores fully autonomously, the LLM required only 5 s.

Beyond time savings, automated approaches may also improve scoring consistency among evaluators. Model assisted clinicians achieved an intraclass correlation coefficient of 0.993 (95% CI of 0.991 to 0.994), compared to 0.968 (95% CI of 0.960 to 0.975) for unassisted evaluators [22]. The most substantial improvements occurred in subjective categories such as mechanical pain and degree of vertebral body collapse. Clinical documentation frequently contains ambiguous descriptions of pain, and radiological reports may lack precise quantitative thresholds for vertebral height loss. Artificial intelligence may help standardise the interpretation of these subjective clinical variables. Transitioning toward objective, semi-automated quantitative models could help to reduce interobserver variability and streamline clinical workflows for patients with spinal metastases.

However, current LLM implementations remain limited by their reliance on textual inputs. These systems process clinical notes and radiology reports, without direct access to the imaging data itself. While these models can improve efficiency and reproducibility, they cannot independently assess morphological features or quantify structural compromise and consequently inherit the subjective biases and potential errors of the source human-authored data. A vision–language framework integrating CT or MRI with clinical documentation could provide a more robust assessment of spinal stability, potentially improving reproducibility, diagnostic confidence, and fracture risk prediction.

## 4. Frontier 1: Biomechanical Modelling and Finite Element Analysis (FEA)

### 4.1. Principles of QCT-Based FEA

Biomechanical modelling, particularly FEA, has emerged as a computational approach for assessing structural integrity and predicting vertebral fracture risk. Traditional clinical scoring systems and dual energy x-ray absorptiometry (DEXA) estimate fragility based on qualitative risk factors or two-dimensional bone mineral density (BMD). In contrast, FEA constructs three dimensional patient specific models that simulate the mechanical behaviour of vertebrae under physiological loads [24,25]. By dividing the vertebral geometry into thousands of smaller structural elements, these models generate detailed maps of internal stress and strain [2].

Instead of assigning uniform material properties across the entire bone, these models can capture the spatial heterogeneity of vertebral microarchitecture. Voxel specific Hounsfield units derived from CT scans are converted into volumetric BMD values, either through the use of calibration phantoms during image acquisition or via phantomless calibration algorithms [7]. These local density values determine the elastic modulus and yield strength assigned to each element using established density to elasticity mathematical relationships [26]. The resulting patient specific models are then subjected to simulated physiological loading conditions, including axial compression, flexion, extension, and lateral bending. By calculating the total reaction force and corresponding displacement at each loading step, the models generate quantitative biomechanical outputs such as whole bone stiffness, yield force, ultimate fracture load, and work to yield. Computational frameworks based on damage mechanics models can simulate the progressive accumulation of structural damage from initial yield to complete vertebral collapse. Multiple investigations have shown that these derived biomechanical parameters classify patients at risk of VCF more accurately than traditional structural or bone density measures, largely because they account for the three dimensional distribution of load bearing cortical and trabecular structures [2,7,25].

### 4.2. Impact of Lesion Characteristics and Failure-Load Patterns

Lytic and blastic metastases alter the mechanical competence of the vertebrae through different mechanisms. Lytic lesions produce localised mechanical weakening through the direct destruction of normal bone; finite element simulations have shown that lytic tumours cause trabecular rarefaction, decrease tissue mineral content, and reduce structural connectivity, leading to an exponential increase in vertebral bulge and a linear reduction in mechanical strength [6,7]. Conversely, blastic metastases are characterised by excessive but irregular formation of new bone. While this process increases local volumetric BMD and trabecular thickness, the newly formed woven bone exhibits disorganised alignment of collagen fibrils and mineral plates. This irregular structure significantly reduces the local Young’s modulus of the tissue, resulting in structurally weak bone that remains highly prone to fracture despite its dense radiographic appearance [27]. In vertebrae containing lytic lesions, large compressive and tensile strains localize directly around the defect. In contrast, vertebrae with blastic metastases display complex strain patterns with heterogeneous strain and tensile concentrations within adjacent healthy tissue. This reflects a compensatory mechanical response to the stiffened blastic bone lesion [15].

Advanced finite element frameworks now integrate distinct constitutive material models to account for these specific pathological alterations; representing an advancement from early studies that modelled metastatic lesions as empty voids [2].

However, metastatic disease often presents with mixed features that complicate structural evaluation. Modern damage mechanics frameworks overcome this limitation by utilizing heterogeneous, voxel by voxel constitutive material assignments to accurately model the complex spatial distribution of both dense blastic woven bone and weakened lytic regions within the same vertebra. This produces high fidelity, element specific material mapping to capture the distinct spatial damage accumulation patterns inherent to mixed lesions. Such approaches have demonstrated reliable predictions of stiffness and strength [28,29].

Besides material properties, anatomic factors further influence the biomechanical response. Transcortical defects and lesions situated near pedicles or costovertebral joints disrupt critical load transfer pathways, reducing overall vertebral strength more severely than small lesions confined entirely to the trabecular centrum [3]. Tumour size is a primary driver of structural instability, particularly in the context of lytic disease. However, relying solely on lesion size provides an incomplete assessment without considering the baseline strength of the unaffected bone matrix. A study examining simulated defects demonstrated that tumour size reduced vertebral strength at a rate 84% faster than it reduced bone density, highlighting that baseline structural strength may have a greater influence than tumour size alone in determining overall fracture risk [7].

### 4.3. Model Assumptions and Methodological Limitations

Biomechanical modelling provides a framework for quantifying how specific metastatic lesion characteristics affect overall vertebral integrity. However, these computational outputs remain highly dependent on underlying model assumptions regarding boundary conditions and methodological limitations should be considered when interpreting computational failure predictions. Clinical translation requires addressing real-world confounders that influence spinal mechanical stability, including posterior element involvement, and prior spinal instrumentation.

The incorporation of posterior elements is critical for accurately simulating physiological stress. Many experimental protocols isolate the vertebral body by excising the posterior structures to simplify testing [7,29]. However, the intact posterior elements, articular facet joints, and posterior ligamentous complexes share a substantial portion of applied spinal loads. One proposed solution for this is mapping posterior structures via three dimensional mesh coordinates [2]. This enables more accurate simulation of multidirectional load transfer pathways during complex physiological movements, such as sagittal flexion, extension, and lateral bending, rather than relying solely on simplified axial compression.

The intervertebral discs play a key role in modulating stress and strain distributions across the vertebral endplates. The degenerative state of these discs significantly alters both the overall mechanical instability and the specific failure patterns of pathologic spines. A growing body of studies have reported approaches for numerical simulation of the effects of intervertebral disc degeneration [30], yet studies in the context of spinal oncology often fail to account for the intervertebral discs. Future works should incorporate the poroelastic properties of the adjacent intervertebral discs to improve predictive accuracy in real clinical settings.

Prior spinal instrumentation presents another major hurdle. Some authors have developed models of the biomechanical effects of virtual pedicle screw insertion on metastatic vertebrae to predict load to failure and evaluate surgical strategies [2]. However, evaluating patients who already possess spinal hardware in vivo remains technically challenging. The actual physical presence of prior metallic spinal instrumentation introduces severe beam hardening and scatter artifacts on routine CT imaging. These metal artifacts artificially distort the local Hounsfield units and subsequently corrupt the critical density to elasticity conversion algorithms. Consequently, this signal scattering creates a significant obstacle for accurate post-surgical finite element modelling, limiting the current applicability of these quantitative tools in instrumented patients.

An important validation gap exists regarding whether computationally predicted damage initiation sites accurately correspond to true clinical fracture morphology. Advanced optical measurement tools offer a method to map experimental strain distributions and validate computational outputs. A 2022 study by Garavelli et al. utilised digital image correlation to validate a subject-specific finite element model of a multi-vertebrae lumbar segment containing a metastatic lesion [31]. The investigators demonstrated a strong correlation between the computationally predicted and experimentally measured surface displacements, yielding determination coefficients (R^2^) exceeding 0.9. Other groups have applied similar optical techniques to characterize metastatic biomechanics. Palanca et al. employed digital image correlation across thirty-five spine segments subjected to complex loading configurations [15]. Their analysis revealed that the size and position of lytic lesions significantly dictated the resulting full-field surface strain perturbations. Moving beyond surface measurements, Fan and colleagues coupled in situ mechanical testing with digital volume correlation to capture three-dimensional internal strain fields [27]. This approach enabled mapping of the distinct mechanical deformations occurring within blastic and lytic trabecular bone under compressive loads. Despite demonstrating high accuracy in isolated cadaveric models, these experimental techniques have not been adequately validated in the clinical context of spinal oncology. Applying digital image and volume correlation requires direct optical access to the prepared bone surface or prolonged high-resolution micro-CT, rendering in vivo clinical studies impossible. Consequently, finite element outputs often remain model-dependent predictions rather than proven clinical realities. Translating these computational tools into routine surgical planning will require extensive prospective clinical validation against documented pathological fracture events to confirm their true diagnostic accuracy.

### 4.4. Logistical Barriers to Clinical Translation

The routine deployment of computational biomechanics in clinical practice requires evaluating its practical feasibility, specifically regarding required inputs, anticipated turnaround times, and computational demands.

FEA traditionally relies on quantitative CT protocols that incorporate a physical calibration phantom placed beneath the patient during image acquisition to accurately convert voxel specific Hounsfield units into volumetric BMD. Integrating physical phantoms into routine clinical imaging workflows, such as standard staging CT or radiotherapy planning scans, presents substantial logistical barriers. A 2024 analysis by Soltani et al. noted that requiring a physical phantom during image acquisition would necessitate the costly and complex recertification of established radiotherapy planning and imaging protocols across different CT and clinical linear accelerator systems [29]. To overcome these barriers, some groups have employed phantomless calibration techniques that utilise internal patient specific anatomical references, such as the known linear attenuation properties of skeletal muscle and adipose tissue, to generate standard calibration curves. For instance, a group at the Mayo Clinic demonstrated strong correlation coefficients (R^2^ of 0.94 to 0.99) between phantom-based and phantom-less estimates of vertebral fracture load thresholds in the context of osteopaenic and osteoporotic spines [32]. Other approaches apply linear correlations assuming a Hounsfield unit of zero corresponds to an apparent density of zero, mapping the maximum measured Hounsfield unit to the accepted maximum density of cortical bone [2].

While phantomless calibration expands the clinical accessibility of computational biomechanics, significant limitations require consideration. Soltani et al. cautioned that although phantomless calibration correlates strongly with phantom based density analysis in general populations, the technique currently lacks rigorous validation within cancer patient cohorts [29]. The structural and densitometric heterogeneity introduced by metastatic disease, such as mixed lytic and blastic lesions, may confound internal reference values. Furthermore, the utility of these phantomless methods has yet to demonstrate reliable generalizability across CT scanner manufacturers. Bianchi et al. similarly acknowledged that while basic phantomless linear mapping allows for comparative FEA, more sophisticated phantomless methods or the use of physical calibration phantoms are necessary to ensure the highest accuracy in defining local bone material properties [2].

Beyond calibration challenges, routine clinical CT possesses lower spatial resolution compared to analyses employing high resolution micro-CT, introducing structural approximations into the finite element meshes. A 2017 study in a cohort of multiple myeloma patients highlighted that clinical scan voxel sizes typically exceed the thickness of the vertebral cortex, which is frequently thinner than 0.5 millimetres [25]. Consequently, finite element models derived from clinical scans suffer from partial volume averaging, where surface elements are assigned material properties representing a mathematical mean of both dense cortical and porous trabecular bone. This averaging effect artificially lowers the simulated cortical modulus while elevating the peripheral trabecular modulus, leading to inaccuracies in predicting load transfer pathways and structural failure [25].

In addition, the computational demands of non-linear FEA may restrict real-time use in busy clinical environments. Highly detailed micro finite element models can require up to two hours to solve on high performance computing clusters [33]. Early attempts to mitigate these limitations have shown promise. An analysis by Stadelmann and colleagues demonstrated that down sampling micro-CT scans to a 1 millimetre isotropic voxel size for FEA model generation (mimicking clinical CT resolution) helped achieve a reasonable processing time of approximately thirty minutes while maintaining structural prediction accuracy [28]. Continued advancements in imaging acquisition techniques such as the use of deep-learning iterative reconstruction methods, and sophisticated deblurring algorithms will be essential for overcoming these limitations and translating computational fracture prediction into a practical clinical tool.

## 5. Frontier 2: Radiomics and Machine Learning in Fracture Prediction

### 5.1. Extracting Sub-Visual Imaging Biomarkers

Radiomics provides a quantitative approach to extracting high-dimensional, mineable data from standard medical images, capturing subtle structural patterns that may not be visible to the human eye. The extraction process typically begins with the segmentation of the target vertebral body or radiation planning target volume on standard CT and MRI. Computational algorithms then extract hundreds to thousands of quantitative features from these regions. These biomarkers fall into several categories, including first-order statistics that describe the distribution of voxel intensities, shape-based metrics, and second-order texture features [8,21]. Second-order features quantify the spatial relationships and heterogeneity of the tissues using mathematical constructs such as the grey level co-occurrence matrix and the grey level run-length matrix. The grey level co-occurrence matrix evaluates how frequently voxel intensities occur next to one another, reflecting tissue heterogeneity, while the grey level run-length matrix measures the length of continuous regions with similar intensity values, capturing patterns of structural uniformity or disruption. To further enhance feature extraction, researchers often apply preprocessing image filters, including wavelet decompositions and Laplacian-of-Gaussian filters. These filters emphasise transitions between regions of different density and highlight the interface between normal trabecular bone and tumour tissue.

### 5.2. Development of Predictive Models

The sheer volume of extracted radiomic features typically exceeds the number of patients in clinical cohorts, creating a substantial risk of model overfitting, meaning the model fits the training data too closely and may not perform well on new patient data. Investigators therefore apply various methodologies for dimensionality reduction, including least absolute shrinkage and selection operator regression to eliminate highly correlated variables and retain features consistently selected across iterations [8]. Other strategies utilise recursive feature elimination to condense thousands of initial parameters into a small predictive subset [21], or filtering techniques that exclude features demonstrating near perfect collinearity and zero importance in gradient boosting models [9]. The retained subset of high-value imaging features is then used to train machine learning models. Various models have demonstrated utility in this domain, including logistic regression, support vector machines, gradient boosting, and artificial neural networks. Among these algorithms, random forest classifiers frequently perform well by combining multiple decision trees to accurately categorise vertebrae at high risk of pathological compression fracture [20,21].

### 5.3. Direct Evidence in Spinal Oncology: Radiomics Versus Morphological Scoring

A growing body of direct evidence from patients with spinal metastases demonstrates that integrated machine learning models significantly outperform conventional morphological criteria in predicting spinal structural failure. A 2025 multi-institutional study by Flores et al. evaluating an all-comer oncological cohort of 977 vertebrae reported that a random forest model using a combined clinical and radiomic feature set yielded superior diagnostic performance [21]. This model achieved an AUC of 0.86 (95% CI of 0.82 to 0.90; *p*-value not reported) with a sensitivity of 78% (95% CI of 70% to 84%) and specificity of 80% (95% CI of 77% to 82%). In contrast, the baseline model relying solely on SINS achieved a significantly lower AUC of 0.75 (95% CI of 0.70 to 0.80; *p*-value not reported).

Similar superiority has been reported in the post-radiation setting. A retrospective analysis by a Johns Hopkins University group combined clinical characteristics with CT and T1-weighted MRI radiomics to predict post-SBRT VCF. The predictive model incorporated body mass index, performance status, total prescription dose and planning target volume dosimetry. This integrated framework achieved an AUC of 0.878 (95% CI of 0.832 to 0.924), a sensitivity of 84.4% (95% CI of 73.3% to 93.3%), and a specificity of 80.0% (95% CI of 74.95 to 85.2%) for predicting fractures following SBRT. This substantially outperformed the standalone morphological scoring model, which yielded an AUC of only 0.579 (95% CI 0.496 to 0.663) alongside a remarkably low specificity of 26.2% (95% CI of 20.8% to 31.7%) [8].

Other investigations evaluating automated quantitative biomarkers in irradiated cohorts have reported comparable improvements. Another retrospective study incorporating dosimetric variables alongside radiomic and clinical features (such as age, gender, follow-up period after treatment, histology, and SINS) showed that a model constructed with all three types of variables achieved an AUC of 0.871 (95% CI and *p* not reported) compared to 0.764 (95% CI and *p* not reported) when utilizing clinical features alone [9]. Quantitative evaluations of specific features show that textural parameters, particularly wavelet-transformed features and metrics of tumour composition, are among the most predictive variables [20,21]. Feature importance analysis in a machine learning study by Gulta et al. identified quantitative lytic and blastic tumour percentages as contributing 21.9% and 17.5% to the predictive weight, respectively, far exceeding the importance of the other SINS parameters of categorical location (contributing 2.2%) or posterolateral involvement (contributing 2.8%) [20]. These algorithms are effective because they quantitatively measure localised bone damage and heterogeneous tumour-related bone destruction, changes that are unlikely to be fully captured by qualitative visual assessment on routine imaging.

### 5.4. Translatability from Osteoporotic Fracture Models

While predictive algorithms designed specifically for spinal metastases are expanding in number, substantial foundational evidence derives from radiomic models originally developed to predict primary osteoporotic fractures. These osteoporotic models provide a useful reference framework for the oncology setting, as both conditions share underlying mechanisms of trabecular microarchitectural degradation and mechanical instability. A longitudinal analysis of 7906 older adults demonstrated that a CT-based radiomics nomogram (a predictive model that combines imaging features into an individual risk score) could accurately predict new osteoporotic vertebral fractures at one, three, and four year intervals [34]. Consistent with the spine oncology literature, this study identified wavelet-transformed features as the strongest predictors of structural failure, supporting the value of these texture-based features in quantifying compromised bone architecture.

In addition, osteoporotic radiomic models demonstrate utility for predicting failures following surgical interventions. A multi-centre investigation developed an MRI-based radiomics nomogram to predict the occurrence of new, adjacent-level vertebral fractures following prophylactic vertebral augmentation [35]. This algorithm integrated radiomic signatures with clinical factors, such as the presence of intravertebral clefts, to achieve excellent diagnostic discrimination with an AUC of 0.886 (95% CI of 0.834 to 0.938; *p*-value not reported). Because prophylactic vertebral augmentation and cementoplasty are frequently utilised to stabilise impending pathological fractures in spinal oncology, similar radiomic nomograms may have potential applications in post-procedural surveillance of patients with metastatic disease.

### 5.5. Challenges and Future Directions in Radiomics

Despite these promising results across both osteoporotic and metastatic populations, several significant limitations warrant caution before widespread clinical implementation can occur. Radiomics models often suffer difficulties maintaining model performance outside of initial datasets. This generally stems from two critical challenges: feature stability related to scanner or protocol heterogeneity, and segmentation variability.

Radiomic feature extraction is sensitive to variations in imaging acquisition technique. Differences in image reconstruction algorithms, scanner manufacturers, tube currents, and slice thicknesses amongst other factors introduce significant protocol heterogeneity. This directly compromises the stability of the extracted features, making it difficult to generalise across institutions. To address these inconsistencies in future multicentre studies, investigators must implement robust feature harmonization techniques. Applying statistical harmonisation tools such as the ComBat algorithm [36] can effectively remove centre-specific batch effects, ensuring that the extracted textural features reflect true biological differences rather than hardware discrepancies.

Segmentation variability represents another potential challenge. The reliance on manual or semi-automated volumetric segmentation introduces a time-intensive bottleneck susceptible to human error and interobserver variability. Translating these predictive models into routine clinical workflows will likely require fully automated segmentation and feature extraction pipelines. Utilizing deep learning architectures, such as convolutional neural networks, can standardise the contouring process to eliminate user-dependent segmentation variations [20,37].

Furthermore, the majority of current radiomics investigations rely on retrospective designs with relatively small cohort sizes, which inherently restricts statistical power and increases the probability of model overfitting. Algorithms must demonstrate sustained diagnostic accuracy across diverse, multi-institutional prospective trials to confirm their generalizability.

In addition to validation hurdles, the “black box” nature of current machine learning classifiers, where their internal logic is not easily interpretable by human evaluators, may reduce clinical trust and hinder adoption in routine practice. Quantifying feature importance could be one practical method to improve interpretability. For instance, a 2025 machine learning analysis by Burgess et al. attempted to overcome the transparency limitations of their predictive tool by explicitly calculating the weight of different clinical, radiographic, and dosimetric factors for post-radiation fracture risk [38]. By identifying the specific variables most relevant to the prediction, model developers allow clinicians to verify that the algorithm relies on biologically plausible risk factors rather than spurious correlations.

## 6. Frontier 3: Microstructural MRI Biomarkers (From Osteoporosis to Metastasis)

### 6.1. The Vertebral Bone Quality (VBQ) Score

T1-weighted MRI sequences are routinely acquired for spinal oncology staging and treatment planning. This specific MRI sequence provides an opportunistic method to assess bone quality without the additional radiation exposure or logistical hurdles associated with dual-energy x-ray absorptiometry. The Vertebral Bone Quality (VBQ) score quantifies bone quality by measuring the median signal intensity of the trabecular bone within selected vertebrae and dividing it by the signal intensity of the cerebrospinal fluid, typically measured at the L3 level [24,39]. In practical terms, this ratio reflects the amount of fatty replacement within the vertebral marrow. Higher signal intensity on these scans reflects greater fatty infiltration and, consequently, degraded bone microarchitecture. Initial applications of this scoring system demonstrated a robust ability to predict osteoporotic fragility fractures, with a retrospective cohort study reporting an odds ratio of 2.40 (95% CI of 1.30 to 4.44; *p* = 0.003) for new incident fractures per one point increase in the score [24].

Translating this metric to the clinical scenario of a patient with spinal metastases, a retrospective analysis of 105 patients with spinal metastases revealed that the VBQ score predicted new VCF independently of traditional SINS morphological criteria [40]. Patients sustaining a new fracture during the observation period demonstrated higher baseline scores than those without a fracture (3.26 versus 2.48; *p* < 0.0001). Utilizing a cutoff score greater than 3.0 yielded a predictive sensitivity of 75.0% (95% CI not reported) and specificity of 85.7% (95% CI not reported), corresponding to an AUC of 0.80 (*p* < 0.0001, 95% CI not reported). Combining this quantitative metric with categorical morphological scoring yielded a predictive accuracy of 89% (*p* < 0.0001, 95% CI not reported) [40].

However, potential limitations exist when applying this technique directly to tumour-infiltrated vertebrae. A subsequent evaluation by Pennington et al. of patients undergoing radiotherapy for mobile spine metastases found that the T1-weighted VBQ score failed to predict pathological fractures. In contrast, CT-derived Hounsfield units less than or equal to 132 remained highly predictive with an odds ratio of 2.53 (95% CI of 1.26 to 5.10; *p* = 0.009) [41]. This discrepancy may partly reflect differences in study design and the biological effects of tumour infiltration, as well as relatively small and heterogeneous sample sizes. Tumour infiltration, peritumoural oedema, and osteosclerosis all reduce the T1 signal intensity within the bone. This reduction can artificially lower the calculated VBQ score, masking underlying structural degradation and potentially mimicking the appearance of normal bone marrow signal. Practically, clinicians should exercise caution when using marrow-based metrics as fracture predictors in extensively tumour-infiltrated vertebrae or those that have recently undergone ablative radiotherapy.

### 6.2. Prognostic Adjuncts: Bone Marrow Adiposity and Paraspinal Atrophy

Chemical shift-encoding MRI provides a more sophisticated, quantitative evaluation of bone marrow composition through the measurement of the proton density fat fraction (PDFF). This technique separates water and fat signals to map the distribution of fat within the trabecular matrix. A longitudinal study by Leonhardt et al. in a cohort of 48 subjects without history of malignancy showed that an accelerated increase in the PDFF significantly preceded the clinical occurrence of incidental VCF [42]. Over a 12-month period, the fat fraction in the fracture group increased by 6.3 ± 3.1%, compared to an increase in only 2.1 ± 2.5% in the non-fracture control group (*p* = 0.03).

Furthermore, in the acute fracture setting, evaluating the ratio of the fat fraction between a fractured vertebra and adjacent normal vertebrae helps predict structural collapse. Yun et al. examined 48 patients with acute osteoporotic fractures and found that individuals exhibiting progressive vertebral collapse demonstrated a significantly lower fat fraction ratio compared to those with stable fractures (0.38 ± 0.13 versus 0.51 ± 0.20; *p* = 0.009). Utilizing a cutoff ratio of 0.42 yielded an area under the curve of 0.723 (95% CI of 0.575 to 0.842; *p* = 0.010) with a sensitivity of 84.0% (95% CI not reported) and a specificity of 60.9% (95% CI not reported) for predicting collapse. This depressed ratio reflects the complex interplay between baseline adiposity and fracture induced local oedema [43].

Similarly, emerging evidence supports paraspinal muscle evaluation as a compelling prognostic adjunct. The structural stability of the spine relies heavily on the support provided by the paraspinal muscles. Reduced muscle mass and increased fat infiltration weaken the ability of these muscles to stabilise the spine, which can impair sagittal balance and shift abnormal mechanical loads onto fragile vertebral bodies. Degeneration of these muscles can manifest on cross-sectional imaging (e.g., CT or MRI) as a reduction in area and an increase in fatty infiltration. A 2023 meta-analysis of osteoporotic populations found that patients with incidental and recurrent VCF exhibited significantly smaller cross-sectional areas of both the erector spinae and multifidus (standardised mean difference −0.575, 95% CI of −0.866 to −0.285, *p* < 0.001) and the psoas muscles (standardised mean difference −0.750, 95% CI of −1.274 to −0.226; *p* = 0.005). The same analysis also reported a significantly greater degree of fatty infiltration in both initial and recurrent fracture cohorts, with standardised mean differences of 0.768 (95% CI of 0.475 to 1.062; *p* < 0.001) and 0.720 (95% CI of 0.258 to 1.182; *p* = 0.002), respectively [44].

While well-validated in osteoporotic cohorts, direct evidence for these methods as fracture risk stratification tools specifically for spinal metastases remains limited. Existing studies have focused on the utility of these metrics in assessing overall mortality and frailty. An artificial intelligence-driven analysis of 143 patients undergoing SBRT for spinal metastases identified osteosarcopenia as the most prevalent musculoskeletal condition, affecting 31% of the studied cohort [37]. In this population, lower psoas muscle density emerged as an independent predictor of post-radiation fracture risk in multivariate models, yielding an odds ratio of 0.968 (95% CI of 0.943 to 0.996; *p* = 0.022). In addition, among male patients, the ratio of psoas muscle volume to vertebral body volume was significantly lower in those experiencing fractures (*p* = 0.0155, d = −0.41). A retrospective study evaluating 196 patients surgically treated for spinal metastases demonstrated that sarcopenia independently predicted an increased risk of one-year mortality, even after controlling for established prognostic tools such as the modified Bauer score, which estimates survival based on tumour type and metastatic burden (hazard ratio 1.58, 95% CI of 1.04 of 2.40; *p* = 0.03) [45].

Integrating quantitative measures of paraspinal muscle volume and quality into routine clinical evaluations could serve to assess overall patient survival and physiological reserve as complementary radiation-free prognostic adjuncts rather than standalone predictors of spinal instability.

## 7. Towards a Multiparametric Paradigm for Spinal Stability

### Integrating the Three Frontiers

Traditional morphological classifications tend to evaluate spinal stability in isolation and rely heavily on subjective visual estimation. Transitioning toward a comprehensive, multiparametric paradigm involves combining quantitative data from biomechanical modelling, radiomic texture analysis, and microstructural imaging markers into integrated predictive models.

Machine learning provides a potential computational framework for this integration, although optimal strategies for combining heterogeneous data types remain to be established. Current predictive models have shown promising performance in retrospective datasets. These combine clinical variables, such as primary tumour histology and the presence of mechanical pain, with high dimensional radiomic features extracted from standard staging CT scans, as detailed in the above sections. Several key studies from each of the three frontiers are summarised in Table A1 found in the Appendix. Importantly, these modalities differ substantially in their level of clinical maturity, with radiomics and MRI biomarkers supported primarily by retrospective clinical data, while biomechanical models often remain computationally intensive and largely confined to research settings.

While combined clinical and radiomic models represent a significant advancement (Table 1, Figure 1), future diagnostic frameworks must expand to incorporate finite element outputs and regional microstructural indices. Vertebral compressive strength and yield load derived from FEA provide direct estimates of vertebral mechanical strength that textural radiomics cannot entirely capture. Similarly, opportunistic MRI measurements of paraspinal muscle cross sectional area and fatty infiltration quantify the supportive muscular structures surrounding the spine.

**Figure 2:**
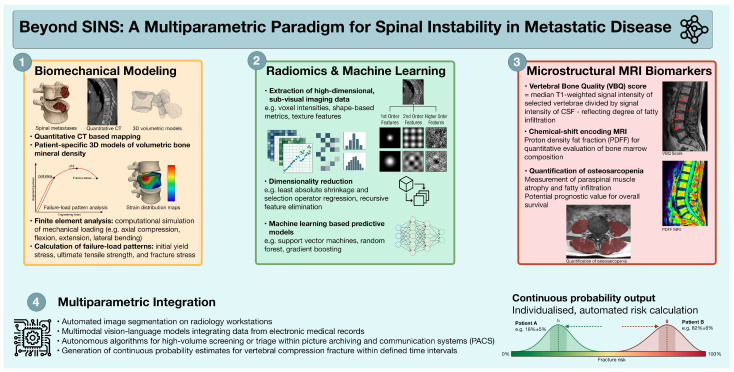
An overview of a multiparametric framework for spinal stability.

By processing these diverse inputs concurrently, an ensemble model could transition fracture risk assessment away from the discrete categorical thresholds utilised by SINS. The integrated model would produce a continuous risk estimate, outputting an individualised probability distribution from zero to one hundred percent predicting the likelihood of structural failure over defined time intervals (Figure 2). Such quantitative risk estimates may provide actionable information during multidisciplinary tumour board discussions and support more personalised treatment planning.

**Figure 3:**
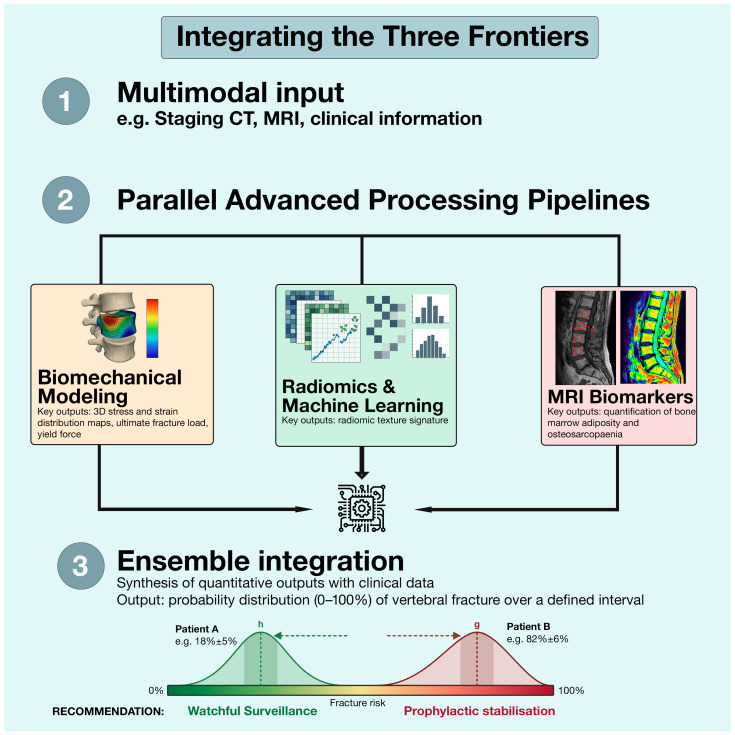
Schematic illustrating a framework for integrating the three frontiers into a multiparametric model.

Within this framework, a hypothetical patient with SINS 9 disease falling in the indeterminate grey zone could undergo further evaluation using parallel processing pipelines receiving standard staging imaging studies as inputs (Figure 3). A high-risk scenario emerges if FEA reveals a failure load falling below physiological thresholds, coupled with a high risk radiomic texture signature. MRI biomarkers signalling an elevated fracture risk such as a high VBQ score and low volumetric paraspinal muscle indices would serve as useful adjuncts. An ensemble deep learning model synthesises these quantitative inputs with extracted clinical information to yield a high continuous fracture probability (e.g., 80% within 12 months). This result would directly inform multidisciplinary management strategies, prompting prophylactic stabilization such as cementoplasty prior to the delivery of ablative SBRT.

In contrast, the model might evaluate a different patient with an identical baseline SINS of 9 but identify stable stress maps on FEA. This could present alongside a low-risk radiomic texture signature and relatively preserved MRI measures of microstructural marrow signal. This combination of normal quantitative biomarkers would result in a low calculated probability distribution of structural failure, reclassifying the patient as stable and supporting safe management with watchful surveillance. This multiparametric approach could resolve the ambiguity of the indeterminate category, sparing stable patients from unnecessary surgical morbidity while ensuring high risk individuals receive timely interventions.

Conceptually, this could resolve the ambiguity of the indeterminate category. Identifying vertebrae at high risk of structural failure before delivery of ablative SBRT allows surgeons to perform targeted prophylactic cementoplasty or percutaneous pedicle screw fixation [46]. Conversely, confirming biomechanical stability in patients with indeterminate morphological scores may prevent unnecessary surgical intervention, sparing vulnerable oncological patients from procedure-related morbidity and prolonged recovery.

## 8. Challenges and Future Directions

### 8.1. Standardisation and Reproducibility

The clinical translation of multiparametric fracture prediction models faces several hurdles related to standardisation and reproducibility. Variations in imaging acquisition protocols, CT reconstruction algorithms, and magnetic field strengths can influence quantitative outputs. For example, a prospective evaluation demonstrated that the predictive threshold for the Vertebral Bone Quality score differed significantly based on the magnetic field strength of the scanner, identifying an optimal cutoff of 3.75 for 1.5 Tesla machines compared to 2.60 for 3.0 Tesla systems [39]. These suggest caution must be applied before transferring proposed thresholds between institutions [39].

As previously discussed, transitioning toward fully automated segmentation pipelines utilizing deep learning architectures will be important to reduce interobserver variability and the time burden associated with manual contouring. Biomechanical frameworks similarly require further refinement prior to broader clinical application. Many current analyses rely on mechanically created defects rather than true pathologically developed metastatic lesions, which oversimplifies the complex biological processes underlying trabecular bone degradation in vivo.

In addition, future models will need to incorporate viscoelastic and rate-dependent material properties to better simulate the multidirectional loading conditions experienced by the human spine. Finally, large multi-institutional prospective studies involving diverse patient populations will be necessary to validate the generalizability of these artificial intelligence and biomechanical models before they can be adopted more broadly in clinical workflows.

### 8.2. Directions for Future Research

Currently, no large-scale validation studies have specifically evaluated the utility of these emerging quantitative methods specifically in cohorts within the SINS 7 to 12 indeterminate grey zone, which represents a critical gap in the published literature. Analyses to date have included specific patient population only indirectly as part of broader mixed cohorts. To truly determine if these emerging computational and microstructural frontiers provide clinically significant incremental value over traditional scoring, future research should explicitly address two concrete research priorities.

First, large-scale prospective validation trials are needed. A key deliverable for these studies will be validating computational predictions directly against objective clinical endpoints, such as actual pathological fracture occurrence, rather than relying on surgical instrumentation rates. Furthermore, future prospective designs should ideally explicitly focus on evaluating these quantitative models within cohorts with indeterminate baseline scores between 7 and 12. Validation of these tools in the clinical grey zone is needed to determine their value as objective triage instruments and to address current evidence gaps.

Second, achieving broad clinical adoption demands rigorous standardisation targets to ensure reproducibility. A measurable deliverable in this domain involves the development and regulatory certification of fully automated segmentation pipelines. Implementing deep learning convolutional neural networks is required to eliminate the time intensive bottleneck and interobserver variability associated with manual image contouring. Researchers must also establish standardised calibration methods that harmonise feature extraction across different scanner manufacturers and magnetic field strengths. For example, the predictive threshold for the Vertebral Bone Quality score fluctuates significantly between 1.5 Tesla and 3.0 Tesla systems, highlighting the need for hardware specific reference ranges prior to broad clinical application.

### 8.3. Clinical Integration

Despite the described clinical potential, significant limitations currently restrict immediate implementation of these integrated predictive tools.

Clinical implementation of these complex models requires seamless integration with radiology workstations and electronic medical records. Automated image segmentation using deep learning convolutional neural networks is essential to eliminate the time intensive bottleneck of manual contouring. Recent open-source deep learning frameworks, such as TotalSegmentator [47,48], demonstrate the feasibility of automated large-scale anatomical segmentation from cross-sectional imaging. These tools may facilitate the integration of quantitative imaging biomarkers into clinical pipelines.

The routine deployment of artificial intelligence algorithms requires navigating complex regulatory and legal landscapes. Most machine learning products related to clinical decision making are presently marketed strictly as support tools rather than autonomous diagnostic systems. This restriction largely stems from stringent regulatory constraints and unresolved liability concerns regarding patient safety and algorithmic errors [49]. Broad clinical adoption will therefore demand the establishment of evidence-based validation, with proper governance and accountability frameworks. Successful integration may also benefit from dedicated educational and training programs for physicians to ensure they can interpret complex algorithmic outputs and recognise potential model biases [22]. Before widespread clinical implementation, health economic analyses are needed to assess the cost-effectiveness, resource use, and impact on treatment timelines of these multiparametric models.

Operational implementation also demands careful consideration of the interaction between human clinicians and artificial intelligence systems. Although collaborative human–AI frameworks are often proposed to maintain clinical oversight, evidence regarding the true efficacy is mixed. An experimental analysis by Agarwal and colleagues involving 277 professional radiologists attempted to evaluate the degree to which radiologists would underutilise or inappropriately override computational input. The analysis showed that this behaviour occasionally resulted in lower diagnostic performance with automated assistance compared to fully autonomous artificial intelligence alone [49]. These findings may reflect cognitive biases such as automation bias, where clinicians over-rely on artificial intelligence outputs, and algorithm aversion, where recommendations may be dismissed altogether.

As a result, the effectiveness of clinical artificial intelligence systems may depend on workflow design rather than solely on model accuracy. Excessive alerting or poorly calibrated thresholds may contribute to alert fatigue, reducing clinician engagement with automated recommendations. Optimal clinical integration may therefore require task-specific deployment strategies, with a human-in-the-loop approach in many scenarios.

Autonomous algorithms may be particularly well suited to high-volume screening or triage tasks. For example, artificial intelligence systems could automatically flag potentially unstable vertebrae within the picture archiving and communication system (PACS), allowing radiologists to prioritise cases while reserving detailed physician review for complex or indeterminate presentations. Providing interpretable outputs, such as visual overlays or quantitative risk estimates, may further enhance clinician trust and facilitate more effective human–AI collaboration.

Future system development will likely rely on multimodal vision–language models capable of simultaneously analysing cross-sectional imaging and unstructured electronic medical records. Such systems could synthesise imaging findings, oncologic history, and clinical symptoms to generate comprehensive automated stability assessments directly at the point of care. Validating these multimodal models for clinical practice will require rigorous prospective trials demonstrating non-inferiority of the model outputs against a panel of subspecialty spinal oncologists and radiologists. These multimodal systems must ultimately be evaluated against documented fracture events to confirm their diagnostic accuracy and clinical value.

## 9. Conclusions

The field of spinal oncology is entering a promising era of precision imaging and personalised risk assessment. While SINS provides an invaluable, standardised framework for multidisciplinary evaluation of spinal instability, its clinical utility is often limited by only moderate interobserver reliability, the ambiguity of the “indeterminate” category (scores 7–12), and limited accuracy for predicting VCF, especially in the context of modern high-dose SBRT. These limitations highlight the need to move “Beyond SINS” toward more quantitative and biologically informed approaches to fracture risk prediction.

To address this gap, this review summarises emerging evidence across three complementary frontiers: computational biomechanics using FEA, artificial intelligence and radiomics derived from routine clinical imaging, and microstructural MRI biomarkers reflecting bone and muscle quality. Each of these approaches captures distinct aspects of spinal stability, including mechanical strength, structural heterogeneity, and the condition of the surrounding musculoskeletal system.

The future of spinal stability assessment is likely to evolve toward a multiparametric framework integrating biomechanical modelling, quantitative imaging biomarkers, and clinical variables. Moving “Beyond SINS” toward such integrated approaches may enable clinicians to shift from categorical scoring systems to individualised estimates of fracture risk, supporting more precise risk stratification and treatment planning for patients with metastatic spinal disease.

## Figures and Tables

**Table 1:** Advantages and Limitations of Conventional Morphological Scoring Versus Emerging Computational and Microstructural Methodologies for Predicting Vertebral Fracture Risk.

Approach	Description	Advantages	Limitations
Spinal Instability Neoplastic Score (SINS)	A standardised consensus scoring system evaluating six clinical and radiographic criteria.	Facilitates multidisciplinary communication and triages cases demonstrating extreme stability or instability.	Uncertain surgical indications for intermediate scores. Poor sensitivity and specificity for predicting VCF following SBRT.
Quantitative Computed Tomography-Based Finite Element Analysis (QCT-FEA)	Computational biomechanical modelling converting voxel specific Hounsfield units into bone mineral density to simulate physiological loading.	Delivers patient specific assessments of mechanical competence and accounts for the complex spatial distribution of bone damage.	Traditionally requires a physical calibration phantom with high resolution micro-CT scanning, which is not feasible in clinical practice. Phantomless models and those derived from clinical resolution scans show promise, but need further validation.
Radiomics and Machine Learning Models	Algorithmic extraction of quantitative textural, intensity, and morphological features from routine clinical imaging.	Significantly outperforms conventional morphological scoring in predicting post-radiation fractures by capturing subtle structural alterations that may not be visible to human eye.	Sensitive to varying imaging protocols and segmentation error and requires improved harmonisation technique and deep learning architecture. Risk of algorithmic overfitting. Requires further external validation before clinical deployment.
Magnetic Resonance Imaging (MRI) Microstructural Biomarkers	Opportunistic quantitative evaluation of bone quality, including the Vertebral Bone Quality score and proton density fat fraction mapping.	Evaluates bone marrow microstructural features as a surrogate for trabecular deterioration without additional ionizing radiation. Evaluates paraspinal musculature providing indicators of both local spinal stability and overall patient frailty.	Tumour infiltration and peritumoural oedema reduce T1 signal intensity, masking structural degradation. This is more well-validated in the context of osteoporotic VCF. Only some studies conducted in spinal oncology patients, but more validation studies are necessary.

## Data Availability

Not applicable.

## Abbreviations

The following abbreviations are used in this manuscript:

CT	Computed Tomography
SINS	Spinal Instability Neoplastic Score
MRI	Magnetic Resonance Imaging
SBRT	Stereotactic Body Radiation Therapy
FEA	Finite Element Analysis
BMD	Bone mineral density
LLM	Large Language Model
VBQ	Vertebral Bone Quality
PDFF	Proton Density Fat Fraction

## Appendix A

**Table A1:** Key Studies in Multiparametric Assessment of Vertebral Fracture Risk.

Study	Study Design	Sample Size	Fracture Endpoint	Main Findings	Key Limitations
**Biomechanical Modelling and Finite Element Analysis (FEA)**					
Fereydoonpour et al., 2025 [7]	QCT-FEA in cadaveric spines	9 cadaveric vertebral bodies with varying tumour sizes (0%, 20%, 35%, and 50%)	The fracture point was identified as the point where a significant drop in measured force occurred	Results demonstrated a strong correlation between experimentally measured and computationally predicted failure forces (r = 0.97, *p* < 0.001) and BMD values (r = 0.96, *p* < 0.001). Tumour size reduced vertebral strength at a rate 84% faster than bone density (*p* = 0.009), suggesting that relying solely on tumour size for fracture risk prediction may be insufficient.	Artificially mechanically induced defects may not fully capture the complex biological and structural heterogeneity of true metastatic lesions. Reliance on a small cadaveric sample subjected solely to compressive loading.
Soltani et al., 2024 [29]	QCT-FEA in cadaveric spines	10 cadaveric metastatic vertebrae containing lytic, blastic, or mixed bone metastases	Until either vertebral failure was registered or the built-in load cell maximum force (15 kN) was reached	A global calibration model explained 83 percent of the measured strength variance (R^2^ = 0.83; *p* = 0.0002) and 90 percent of the measured stiffness variance (R^2^ = 0.90; *p* < 0.0001) across lytic, blastic, and mixed lesions. Utilizing a finer 1 mm mesh size localised damage accumulation in low density regions. The FE damage-based simulations highlighted the differences in the pattern of spatial damage evolution between blastic and lytic vertebral bodies.	Experimental protocol relied on isolated cadaveric vertebral bodies subjected to monotonic uniaxial compression. Removing the posterior elements and intervertebral discs simplified the boundary conditions limiting generalisation to the dynamic loading environment experienced in vivo and presenting a hurdle for clinical application.
Campbell et al., 2017 [25]	Cross-sectional study evaluating QCT-FEA derived from clinical low-dose CT scans of multiple myeloma patients	Single (T12) vertebra of 104 multiple myeloma patients, 45 with fracture and 59 without	Not applicable. Known fractures diagnosed on CT	The computational outputs showed good ability to discriminate between patients with and without VCF. Patients with fracture showed lower vertebral stiffness (–15.2%; OR = 1.73; 95% CI of 1.11 to 2.70; *p* = 0.010), yield force (–21.5%; OR = 2.09; 95% CI of 1.27 to 3.43; *p* = 0.002;), and work to yield (–27.4%; OR = 2.28; 95% CI of 1.33 to 3.92; *p* = 0.001;) compared to nonfracture patients.	Protocol assumed isotropic material properties and simulated only static axial compression. Low spatial resolution (1.5 mm slice thickness) of the routine clinical imaging used for model generation, potentially resulting in partial volume averaging effects.
Palanca et al., 2021 [15]	QCT-FEA in cadaveric spines	16 spines of active donors with a history of spinal metastasis. A total of 35 metastatic vertebrae, were extracted	Spine segments were not loaded to failure. Instead, the loading end-point was defined as approximately −3000 microstrain	Strain distributions were verified with digital image correlation to measure the full-field strain distribution across the vertebral surfaces. Lytic metastases demonstrated critical deformations, yielding an average twofold increase in strain compared to controls, with localised peaks reaching a fourteenfold increase. Conversely, vertebrae containing blastic or mixed lesions exhibited strain deformations similar to or lower than the healthy control segments. Among lytic lesions, the size of the lesion was significantly correlated with the perturbation to the strain distribution (R^2^ = 0.72; *p* < 0.001). The SINS showed poor correlation with the percentage strain difference, and only in case of lytic lesions (R^2^ = 0.25, *p* < 0.0001).	The non-destructive loading protocol prevented the determination of ultimate fracture loads.
**Radiomics and Machine Learning in Fracture Prediction**					
Flores et al., 2025 [21]	CT radiomics combined with clinical features	Multi-institutional study 164 patients (977 thoracolumbar vertebrae)	Both progressive pre-existing and new-onset fractures	Selected clinical features included SINS, SINS component for <50% vertebral body collapse, SINS component for “none of the prior 3” (i.e., “none of the above” on the SINS component for vertebral body involvement), histology, age, and BMI. Of the 2015 radiomic features, recursive feature elimination selected 19 to be used in the pure radiomic model and the combined clinical-radiomic model. A random forest classifier integrating clinical variables and radiomic features was the best performing model achieved an AUC of 0.86 (95% CI of 0.82 to 0.90), sensitivity of 78% (95% CI of 70% to 84%), and specificity of 80% (95% CI of 77% to 82%). This integrated approach outperformed the baseline SINS model, which only achieved an AUC of 0.74 (95% confidence interval 0.69 to 0.79).	The number of instances (N) is relatively low for machine learning techniques. The reliance on a retrospective cohort predominantly from a single institution introduces a risk of algorithmic overfitting and necessitates external multi-institutional validation.
Gui et al., 2022 [8]	CT and T1-weighted MRI radiomics combined with clinical features	95 vertebral levels treated with SBRT	Any new vertebral body height loss or progression of an existing fracture prior to local progression	Selected clinical features included BMI, performance status, total prescription dose, dose to 99% of the planning target volume, lumbar location, and 2 components of the Spine Instability Neoplastic Score (SINS): lytic tumour character and spinal misalignment. Selected radiomic features included 5 features from CT and 3 features from MRI. The best performing model was a random forest model, using a combination of selected clinical and radiomic features, achieved an area under the curve of 0.878 (95% confidence interval 0.832 to 0.924), a sensitivity of 84.4% (95% CI of 73.3% to 93.3%), and a specificity of 80.0% (95% CI of 74.95 to 85.2%). This performed significantly better than models relying solely on conventional clinical triage components, which yielded an AUC of 0.579 (95% CI 0.496 to 0.663).	The relatively small sample size derived from a single institution increases the risk of overfitting and necessitates external multi-institutional validation.
Gulta et al., 2024 [20]	CT radiomics combined with clinical features	179 patients (300 thoracolumbar segments)	Both progressive pre-existing and new-onset fractures	This study used CT imaging biomarkers derived from SBRT treatment planning imaging and machine learning models to predict VCF following SBRT. A random forest algorithm achieved a balanced accuracy of 0.700, representing a 36 percent improvement over the 0.517 balanced accuracy yielded by manual morphological assessment. Feature importance analysis identified coronal angle (0.227) and lytic percentage (0.219) as the strongest predictors of structural failure.	The dataset featured a significant class imbalance, with post-radiation fractures occurring in only 18.3 percent of cases, requiring synthetic minority over-sampling techniques to mitigate this imbalance. Small data set also means that external validation across diverse patient populations is needed.
Seol et al., 2023 [9]	Radiation therapy planning CT radiomics combined with clinical features	85 patients (with total of 114 spinal lesions with 21 VCF)	New-onset fractures post SBRT	The model that used all features (radiomics, clinical, and dosimetric) showed the highest performance with an AUC of 0.871. while the models using only clinical or clinical and radiomics features had lower AUC values of 0.764. However, the radiomics and dosimetric model had the highest accuracy of 0.829 and the highest F1-score of 0.650, while the model combining all three types of features had a lower F1-score of 0.573.	Small sample data which would need external validation.
**Microstructural MRI**					
Ehresman et al., 2020 [40]	Evaluated T1-weighted MRI VBQ	105 patients (56 patients with VCF)	New VCF in patients with spinal metastases	On univariable analysis, the factors associated with new VCF were smoking status, steroid use longer than 3 months, the SINS, and the novel scoring system—the VBQ score. On multivariable analysis, only the SINS and VBQ score were significant predictors of new VCF and, when combined, had a predictive accuracy of 89%. The VBQ score was significantly higher in patients experiencing incident fractures compared to those without fractures (3.50 ± 0.78 versus 3.01 ± 0.69; *p* < 0.001). The VBQ score was shown to have excellent interrater reliability with an ICC of 0.899. Finally, the combined categorical SINS and binary VBQ score demonstrated the highest AUC of 0.89.	The retrospective design lacked direct correlation with dual energy x-ray absorptiometry data for all patients, and a correlation could not be tested with VBQ score. Another limitation is the potential for missed fractures, since the average follow-up time was less than 3 years.
Leonhardt et al., 2022 [42]	Evaluated PDFF and T2*	48 patients (12 with incidental VCF, 36 controls)	Incidental VCF without evidence for a high-energy traumatic or malignant aetiology	Prior to the occurrence of an incidental VCF, PDFF in vertebrae increased in the VCF group (PDFF change = 6.3 ± 3.1%) and was significantly higher than the change in PDFF in the group without VCF (PDFF change =2.1 ± 2.5%; *p* = 0.03). When assessing the differences in rates of change in patients that develop incidental fractures, there was a significant change in slope for PDFF (2.32 per 6 months, 95% CI of 0.31 to 4.32; *p* = 0.03), but not for T2* (0.02 per 6 months, 95% CI of 0.98 to 0.95; *p* = 0.90) or BMD (−4.84 per 6 months, 95% CI of −23.4 to 13.7; *p* = 0.60).	The relatively small number of fracture events limits statistical power, reflecting the stochastic nature of incidental fractures in patients not explicitly identified as high risk. Another limitation is the incomplete data set for BMD measurements using QCT; as the patients already received MR examinations and there was often no justifying indication for CT.
Pennington et al., 2024 [41]	Compared Hounsfield Units (HUs) and VBQ in mobile spine metastases treated with radiotherapy	100 patients (35 had post RT pathologic fractures)	Radiographically documented VCF post-SBRT	On univariable analysis, the mean HUs of the vertebrae adjacent to the fractured vertebra were significantly lower among those experiencing fracture Cox regression found that HUs ≤ 132 (OR 2.533, 95% CI of 1.257 to 5.103; *p* = 0.009), ≥3 WBB vertebral body segments involved (OR 2.376, 95% CI of 1.132 to 4.987; *p* = 0.022), and axial pain (OR 2.036, 95% CI of 0.916 to 4.526; *p* = 0.081) predicted increased fracture odds, while prostate pathology predicted decreased odds (OR 0.076, 95% CI of 0.009 to 0.613; *p* = 0.016). Sensitivity analysis suggested that an HU threshold of ≤132 and a SINS of ≥7 identified patients at increased risk of fracture. VBQ was not significantly associated with fracture odds.	Retrospective study with small sample size. Another limitation stems from the use of a modified VBQ score, which places the cancellous bone ROIs in the vertebrae immediately cephalad and caudad to the pathologic/treated segment. This is different from the L1–4 VBQ score originally described; nevertheless, it was used to better assess the locoregional bone quality, similar to the assessment made using HU.
Yun et al., 2021 [43]	Evaluated PDFF	48 patients with osteoporotic VCF	Acute VCF without associated tumour, infection, or ruptured disc	The ratio of the proton density fat fraction between a fractured vertebra and an adjacent normal vertebra was significantly lower in patients exhibiting progressive structural collapse (0.38 ± 0.13 versus 0.51 ± 0.20; *p* = 0.009), whereas the difference in the PDFF itself was not statistically significant. The optimal cutoff value of the PDFF ratio for predicting OVCF progression was 0.42; this threshold corresponded to sensitivity, specificity, and accuracy values of 84.0%, 60.9%, and 72.9%, respectively, with an area under the curve of 0.723 (95% CI of 0.575 to 0.842; *p* = 0.10).	Single centre retrospective evaluation with a limited sample size. Varied criteria to define VCF progression was used. Patients with multilevel or old compression fractures were excluded.
